# 4113 Infusing a CTSA Program with Causal Pathway Thinking to Transform Evaluation from Operations to Impacts

**DOI:** 10.1017/cts.2020.240

**Published:** 2020-07-29

**Authors:** Rhonda G Kost, Leslie Boone, Sarah Cook, Sarah Nelson, Consuelo Hopkins Wilkins, Mary Stroud, Leah Dunkel, Loretta Byrne, Michelle Jones, Paul A. Harris, Roger Vaughan

**Affiliations:** 1 Rockefeller University; 2 Vanderbilt University Medical Center

## Abstract

OBJECTIVES/GOALS: Innovations with positive health impact are a high priority for NCATS and CTSAs. Program design that uses the Causal Pathway approach incorporates performance indicators that assess impact. We applied Causal Pathway thinking to an ongoing national program to enhance the evaluation of program impact. We report Lessons Learned. METHODS/STUDY POPULATION: We conducted a day-long onsite workshop to introduce the model to the project team, build capacity, and map the existing program elements to Logic Models representing program Specific Aims. A local Causal Pathway (CP) champion was identified. Alignment of the Logic Models with the CP approach (input→activities→ outputs→effects/impact) developed iteratively through biweekly, then monthly conferral among stakeholders. Key tasks included distinguishing among activities, outputs, and effects (impacts), and identification of performance indicators for each stage of the Causal Pathway. Visualization tools and an additional late stage half-day workshop were used to foster consensus. Implementation of the CP model tested the feasibility of collecting specific indicators and prompted model revisions. RESULTS/ANTICIPATED RESULTS: Program leadership and team members (n = 30) participated in the kick-off workshop. Four Specific Aims were mapped to Logic Models. Multiple Causal Pathway (CP) diagrams, one for each project in the program, were developed and mapped to Aims. Alignment of CP threads to Aims and identification of performance indicators required iteration. CP threads converged onto common final Impacts, sometimes crossing to another Aim. Performance indicators for operations were readily accessible to team members, and less so for impacts. Assumptions about program effects were subjected to specific indicators. Over time, Leadership noticed more expression of CP thinking in daily activities. New projects developed during this period incorporated the CP approach. Teams were able to streamline and simplify Logic/CP models. DISCUSSION/SIGNIFICANCE OF IMPACT: Through capacity-building and mentored exercises, an innovation team was able to infuse CP thinking into the evaluation of their ongoing program. The CP approach to design and evaluation maps progress and indicators across the life of a program from initial activities to its ultimate impact.

